# Incidence of respiratory depression between ciprofol and propofol after anesthesia: A systematic review and meta-analysis

**DOI:** 10.1097/MD.0000000000040037

**Published:** 2024-10-11

**Authors:** Jinfang Zeng, Qian Cao, Aonan Hong, Zhen Gu, Jinjin Jian, Xiao Liang

**Affiliations:** a Department of Anesthesiology, Wuxi No. 2 People’s Hospital (Wuxi Clinical College of Nantong University, Jiangnan University Medical Center), Wuxi, China; b Department of Anesthesiology, Affiliated Hospital of Nanjing University of Chinese Medicine, Jiangsu Province Hospital of Chinese Medicine, Nanjing, China; c Department of Anesthesiology, Affiliated Hospital of Jiangnan University, Wuxi, China.

**Keywords:** ciprofol, meta-analysis, propofol, respiratory depression

## Abstract

**Background:**

Respiratory depression is a common complication during operative procedures, meanwhile, ciprofol can provide effective sedation for surgical operations. However, there is not enough evidence to prove the advantage of ciprofol in reducing respiratory depression. So, this meta-analysis aimed to assess the efficacy of ciprofol on the incidence of respiratory depression compared with propofol.

**Methods:**

Two individual researchers conducted searches for randomized controlled trials in PubMed, Embase, and Cochrane Central Register of Controlled Trials. The meta-analysis was performed using Review Manager software.

**Results:**

Seven trials with a total of 1408 patients were included in this meta-analysis. The results showed that ciprofol could reduce the incidence of respiratory depression compared with propofol (risk difference [RD] = −0.09, 95% confidence interval [CI]: −0.15 to −0.04). Ciprofol significantly reduced the incidence of respiratory depression when the method of administration was intravenous injection (RD = −0.06, 95% CI: −0.10 to −0.03), or continuous infusion (RD = −0.30, 95% CI: −0.45 to −0.15). Meanwhile, ciprofol significantly reduced the incidence of respiratory depression with the dosage not only greater than or equal to 0.4 mg/kg (RD = −0.11, 95% CI: −0.20 to −0.02), but also <0.4 mg/kg (RD = −0.08, 95% CI: −0.13 to −0.02). And ciprofol significantly reduced the incidence of hypoxemia (risk ratio [RR] = 0.47, 95% CI: 0.28 to 0.80), injection pain (RD = −0.32, 95% CI: −0.46 to −0.17), body movement (RR = 0.60, 95% CI: 0.43 to 0.84), dizziness (RR = 0.75, 95% CI: 0.62 to 0.90). Finally, ciprofol did not increase awakening time (standard mean difference [SMD] = 0.15, 95% CI: −0.02 to 0.31).

**Conclusion:**

From this meta-analysis, it is demonstrated that ciprofol might reduce the incidence of respiratory depression and injection pain. These benefits are important in surgery to ensure safe and rapid postoperative recovery. So, ciprofol may be a safe and appropriate drug with fewer adverse effects used in clinical anesthesia.

## 1. Introduction

As one of the most commonly identified complications after general anesthesia, respiratory depression can increase pulmonary complications and prolong hospitalization, even leading to mortality.^[1–3]^ Therefore, one of the most undesired conditions for patients is respiratory depression, especially if the patient is elderly or suffers from multiple basic diseases.

With the rapid development of science and the promotion of comfortable medical treatment, more operations and examinations are being carried out under general anesthesia, especially painless endoscopy. As one of the most frequently used intravenously anesthetics, propofol has a rapid onset of action, short half-life, high removal rate, high tolerance, absence of accumulation, rapid response, and other pharmacokinetic properties.^[4–6]^ However, it is characterized by limitations such as a high incidence of respiratory depression, injection pain, allergic shock, suppression of circulatory function, narrow treatment window, lack of availability of antagonists, infusion complications, especially in patients with impaired cardiac performance, such as the elderly and with cardiac disease.^[7–10]^ Meanwhile, patient satisfaction and comfort levels are reduced. It is worth remarking that there has been a continuing search for sedatives with better sedation and fewer side effects, especially for aging patients and higher-risk patients.

Ciprofol (HSK 3486) is the latest 2, 6-disubstituted phenol derivative independently developed in China, which can increase the inward flow of chloride ions mediated by gamma-aminobutyrate (GABA) receptors, leading to central nervous system inhibition, thus achieving sedation or anesthesia, and binds more tightly to A-type aminobutyric acid (GABAa) receptors compared with propofol.^[11]^ In addition, it has a similar chemical structure to propofol and has improved pharmacological and physicochemical properties compared to propofol.^[12]^ It is distinguished by a clear process of assimilation, delivery, metabolism, and excretion, a low incidence of hypotension and respiratory depression, and a high level of safety.^[13]^ Meanwhile, as a new intravenous anesthetic with a significant sedative effect, the pharmaceutical effect of ciprofol is up to 5 times more potent than propofol, which means that only 20% of the dose of ciprofol is needed to achieve the same anesthesia effect as propofol.^[14]^ Clinical studies have proven that ciprofol can be safely used for sedation in gastrointestinal endoscopy and general anesthesia. A phase I study involving healthy Chinese participants showed that 0.4 to 0.9 mg/kg of ciprofol was well tolerated, with a quick onset of effects and a rapid recovery.^[15]^

To our knowledge, no quantitative analysis was done for the combination of related data primarily for the incidence of respiratory depression between ciprofol and propofol after anesthesia. Therefore, we proceeded with the present meta-analysis to explore the efficacy and safety of ciprofol on respiratory depression.

## 2. Methods

We conducted a meta-analysis to assess the incidence of respiratory depression of ciprofol and propofol after anesthesia as recommended by the PRISMA statement. The registration number of the study in PROSPERO is CRD42023467562.

### 2.1. Search approach and eligibility standards

The Cochrane Library, Embase, and PubMed databases were systematically searched by Z.J.f. and L.X. for studies related to ciprofol, propofol, and respiratory depression. The search was conducted through August 26, 2024, and there were no language restrictions. In addition, the reference lists of original reports, case reports, and reviews were checked to identify.

### 2.2. Research selection

Data search included author name, publication year, anesthesia, and surgery type/duration, interventions, cases of respiratory depression, and total patients. Two authors (G.Z. and J.J.J.) independently assessed articles for inclusion/exclusion criteria, with any disputes discussed by all authors.

### 2.3. Inclusion criteria

Studies were included if they met all eligibility criteria, stated as (1) population: adult patients (age ≥ 18 years, male or female, with body mass index 18–30 kg/m^2^_._) undergoing surgery or painless examination under general anesthesia or intravenous anesthesia; (2) intervention: ciprofol; (3) comparator: propofol alone. If the control group was included in the article which compared ciprofol versus other anesthetics, these articles would be excluded, (4) primary outcomes: the incidence of respiratory depression between ciprofol and propofol; secondary outcomes: the incidence of hypoxemia, injection pain, body movement, dizziness, hypotension, bradycardia, postoperative nausea and vomiting (PONV), and awakening time. (5) study types: randomized controlled trials.

### 2.4. Exclusion criteria

1. registration number or abstract only, reviews, nonclinical studies, and case observations;
2. not randomized controlled trials;
3. missing data; reduplicated studies;
4. the experimental group and the control group were not compared with propofol and ciprofol;
5. incorrect statistical analysis; improper outcome measures;
6. meta-analysis, case reports, editorials, and meeting abstracts.

### 2.5. Information extraction and evaluation of bias risk

Two authors (H.A.N. and G.Z.) independently assessed study quality using Cochrane Collaboration guidelines. Six categories (random sequence generation, blinding, allocation concealment, incomplete outcome data, selective reporting, and other bias) were evaluated, with the first 3 categories considered “key areas.” Each category was classified as high risk, unclear risk, or low risk. Bias risk was assessed based on 3 key areas: high (high risk of bias in 1 or more key areas). “unclear” (unclear risk of bias in 1 or more key areas) and “low” (low risk of bias in all key areas).

### 2.6. Quality analysis of evidence

The quality of evidence was evaluated by the GRADE (Grades of Recommendation, Assessment, Development, and Evaluation) system using the Guideline Development Tool.

### 2.7. Outcome measures

The incidence of respiratory depression between ciprofol and propofol, the incidence of injection pain, bradycardia, and PONV were estimated by calculating pooled risk difference (RD), the incidence of hypoxemia, body movement, dizziness, and hypotension were estimated by calculating pooled risk ratio (RR), the awakening time was assessed by pooled standard mean difference (SMD), with 95% confidence intervals (CI). The overall effect was determined by the Z test (*P* < .05) and was considered statistically significant. A fixed effects model was adopted when I^2^ ≤ 50%, otherwise, a random effects model was used. Sensitivity analysis was performed to test the robustness of these results, by reanalyzing the data of low-risk and unclear-risk studies only. Subgroup analyses were based on the method of administration, type of operation, and dosage of administration.

## 3. Results

### 3.1. Study selection

**Figure 1.**
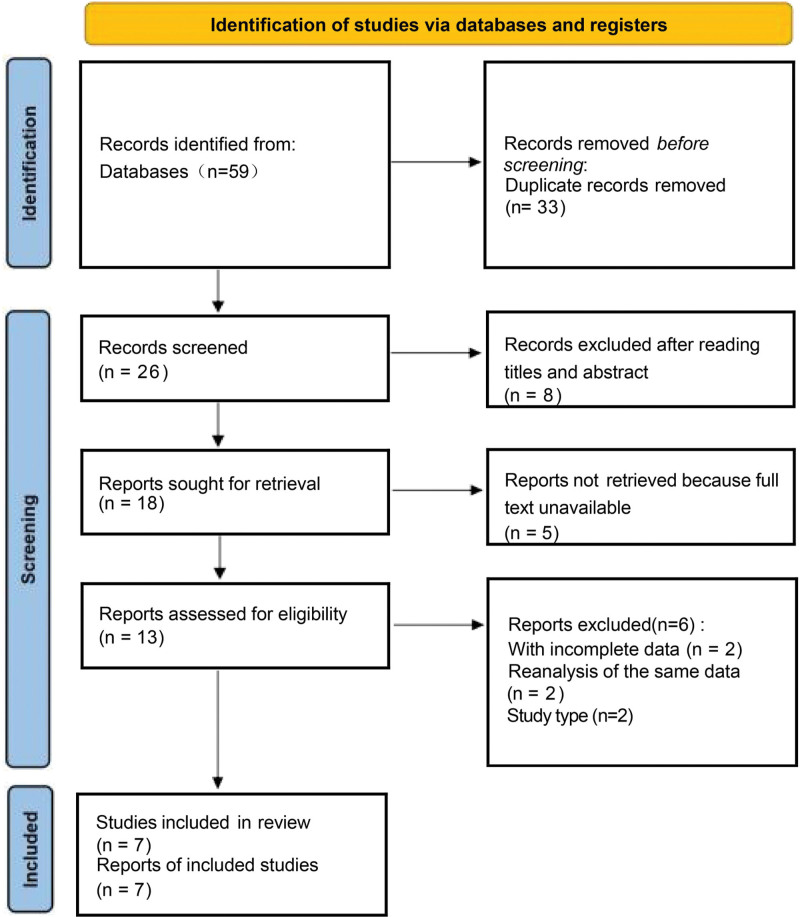
Flow diagram of the inclusion and exclusion process.

As shown in the flow diagram (Fig. 1), the search of PubMed, Embase, Cochrane Library, and reference lists yielded 59 articles. Initially, 8 trials were discarded because they were not controlled trials by reading the titles. Then, 33 trials were excluded for duplicates and 4 were reviewed. Then, 5 trials did not satisfy the inclusion. Thirty papers were carefully read, and we found no related endpoints were reported in 6 papers, so they were excluded. Finally, 7 trials^[16–22]^ that met the selection criteria were included in the meta-analysis.

### 3.2. Study characteristic

Of all the included studies, 7 trials^[16–22]^explored the incidence of respiratory depression of ciprofol and propofol (Table 1). All the included documents are from 2022 and later. There are gastrointestinal endoscopies of 1243 cases. The number of cases of the dosage of ciprofol is more than or equal to 0.4 mg/kg was 1061 cases and <0.4 mg/kg was 347 cases. Moreover, the number of cases when the method of administration is intravenous injection was 1243 cases, and continuous infusion was 165 cases.

**Table 1:** General information of patients with incidence of respiratory depression.

Author	Year	Age	Sex (male/female)	Comparisons (group)	Operation	Respiratory depression	Total
Chen, L.^[18]^	2023	18–80 years	22/16	Ciprofol 0.2 mg/kg	Gastrointestinal Endoscopy	0	38
			12/24	Ciprofol 0.3 mg/kg		0	36
			11/20	Ciprofol 0.4 mg/kg		4	31
			18/26	Propofol 1.5 mg/kg.		4	44
Gao, S. H.^[21]^	2024	≥18 years	34/48	Ciprofol 0.4 mg/kg	Colonoscopy	2	82
			32/50	Propofol 2.0 mg/kg		11	82
Hu, C.^[16]^	2021	18–49 years	–	Ciprofol 0.4 mg/kg maintenance 0.4 mg/kg/h	Healthy subjects	1	8
			–	Propofol 2 mg/kg maintenance 2 mg/kg/h		5	8
Lan, H.^[19]^	2022	18–70 years	0/75	Ciprofol 0.4 mg/kg maintenance dosage of 0.6–1.2 mg/kg/h	Hysteroscopy	3	75
			0/74	Propofol 2.0 mg/kg and then maintained at 3.0–6.0 mg/kg/h		23	74
Li, J.^[17]^	2022	18–65 years	55/89	Ciprofol 0.4 mg/kg	Gastroscopy and colonoscopy	4	144
			63/82	Propofol 1.5 mg/kg		8	145
Liao, J.^[20]^	2023	18–65 years	87/98	Ciprofol 0.4 mg/kg + sufentanil 0.05 µg/kg	Gastroscopy	14	185
			77/106	Propofol 2 mg/kg + sufentanil 0.05 µg/kg		31	183
Zhang, J.^[22]^	2023	18–65 years	–	Ciprofol 0.3 mg/kg + 7 μg/kg alfentanil	Bidirectional endoscopy	8	93
			–	Propofol 1.2 mg/kg + 7 μg/kg alfentanil		13	92

### 3.3. The methodological quality of the included studies

**Figure 2.**
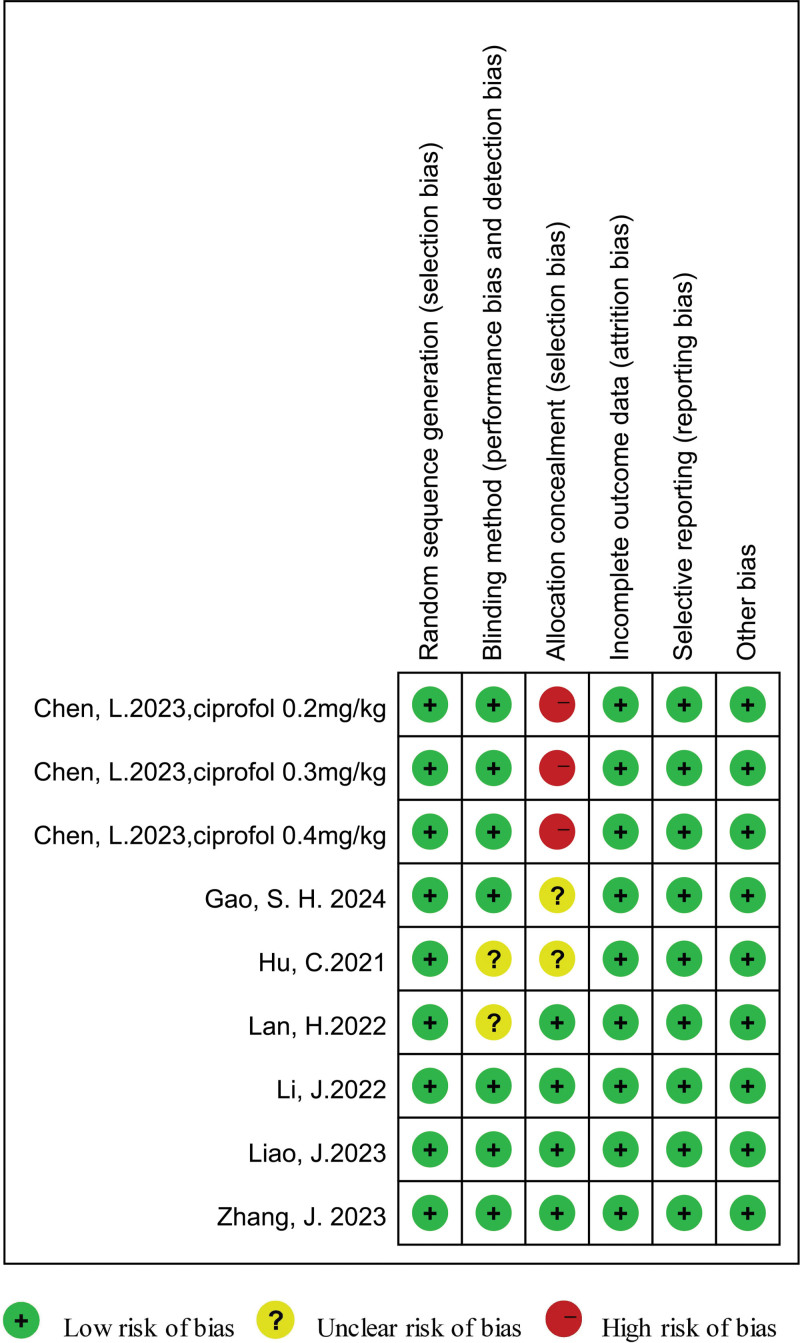
Summary of the risk of bias of the included studies.

All trials^[16–22]^provided a detailed description of randomization. Five^[17,18,20–22]^ studies were double-blinded; 4^[17,18,20,22]^ reported allocation concealment. All the studies^[16–22]^had no complete outcome (attrition bias) and all the studies^[16–22]^ reported all the endpoints mentioned in Section 2 (reporting bias). Other biases might exist in all trials.^[16–22]^ An overview of the risk of bias is summarized in Figure 2.

### 3.4. Quality of evidence

GRADE system grades of evidence showed that having a serious risk of bias in some of those studies, and the total number of events is <300, and RR < 0.5. All studies were designed with randomized mothed, quality of efficacy of respiratory depression between ciprofol and propofol after anesthesia was evaluated as moderate evidence. And most qualities of efficacy of hypoxemia, dizziness, body movement, bradycardia, etc between ciprofol and propofol after anesthesia was evaluated as the low evidence (Table 2).

**Table 2:** GRADE summary between ciprofol and propofol.

Quality assessment							No. of patients		Effect		Quality	Importance
No. of studies	Design	Risk of bias	Inconsistency	Indirectness	Imprecision	Other considerations	Respiratory depression	Control	Relative (95% CI)	Absolute		
Respiratory depression												
9	Randomized trials	Serious*	No serious inconsistency	No serious indirectness	Serious†	Strong association‡	36/692 (5.2%)	103/716 (14.4%)	See comment	94 fewer per 1000 (from 40 fewer to 150 fewer)	⊕⊕⊕Ο MODERATE	CRITICAL
								13.4%		87 fewer per 1000 (from 38 fewer to 139 fewer)		

Quality assessment							No. of patients		Effect		Quality	Importance
No. of studies	Design	Risk of bias	Inconsistency	Indirectness	Imprecision	Other considerations	Hypoxemia	Control	Relative (95% CI)	Absolute		
Hypoxemia												
2	Randomized trials	Serious*	Serious§	No serious indirectness	Serious†	Strong association‡	18/329 (5.5%)	38/328 (11.6%)	RR 0.47 (0.28–0.8)	61 fewer per 1000 (from 23 fewer to 83 fewer)	⊕⊕ΟΟ LOW	CRITICAL
								10.9%		58 fewer per 1000 (from 22 fewer to 78 fewer)		

Quality assessment							No. of patients		Effect		Quality	Importance
No. of studies	Design	Risk of bias	Inconsistency	Indirectness	Imprecision	Other considerations	Hypotension	Control	Relative (95% CI)	Absolute		
Hypotension												
6	Randomized trials	Serious*	No serious inconsistency	No serious indirectness	No serious imprecision	None	145/587 (24.7%)	191/584 (32.7%)	RR 0.81 (0.62–1.07)	62 fewer per 1000 (from 124 fewer to 23 more)	⊕⊕⊕Ο MODERATE	IMPORTANT
								45.1%		86 fewer per 1000 (from 171 fewer to 32 more)		

Quality assessment							No. of patients		Effect		Quality	Importance
No. of studies	Design	Risk of bias	Inconsistency	Indirectness	Imprecision	Other considerations	Bradycardia	Control	Relative (95% CI)	Absolute		
Bradycardia												
6	Randomized trials	Serious*	Serious§	No serious indirectness	Serious†	None	37/587 (6.3%)	37/584 (6.3%)	See comment	1 fewer per 1000 (from 30 fewer to 30 more)	⊕ΟΟΟ VERY LOW	IMPORTANT
								5%		0 fewer per 1000 (from 24 fewer to 24 more)		

Quality assessment							No. of patients		Effect		Quality	Importance
No. of studies	Design	Risk of bias	Inconsistency	Indirectness	Imprecision	Other considerations	PONV	Control	Relative (95% CI)	Absolute		
PONV												
5	Randomized trials	Serious*	Serious§	No serious indirectness	Serious†	None	20/383 (5.2%)	30/407 (7.4%)	See comment	24 fewer per 1000 (from 60 fewer to 10 more)	⊕ΟΟΟ VERY LOW	IMPORTANT
								4.6%		15 fewer per 1000 (from 37 fewer to 6 more)		

Quality assessment							No. of patients		Effect		Quality	Importance
No. of studies	Design	Risk of bias	Inconsistency	Indirectness	Imprecision	Other considerations	Injection pains	Control	Relative (95% CI)	Absolute		
Injection pains												
9	Randomized trials	Serious*	No serious inconsistency	No serious indirectness	No serious imprecision	None	23/692 (3.3%)	299/716 (41.8%)	See comment	317 fewer per 1000 (from 171 fewer to 459 fewer)	⊕⊕⊕Ο MODERATE	CRITICAL
								33.7%		256 fewer per 1000 (from 138 fewer to 371 fewer)		

Quality assessment							No. of patients		Effect		Quality	Importance
No. of studies	Design	Risk of bias	Inconsistency	Indirectness	Imprecision	Other considerations	Body movement	Control	Relative (95% CI)	Absolute		
Body movement												
2	Randomized trials	Serious*	Serious§	No serious indirectness	Serious†	None	37/260 (14.2%)	61/257 (23.7%)	RR 0.6 (0.43–0.84)	95 fewer per 1000 (from 38 fewer to 135 fewer)	⊕ΟΟΟ VERY LOW	NOT IMPORTANT
								34.4%		138 fewer per 1000 (from 55 fewer to 196 fewer)		

Quality assessment							No. of patients		Effect		Quality	Importance
No. of studies	Design	Risk of bias	Inconsistency	Indirectness	Imprecision	Other considerations	Dizziness	Control	Relative (95% CI)	Absolute		
Dizziness												
5	Randomized trials	Serious*	No serious inconsistency	No serious indirectness	Serious†	None	93/512 (18.2%)	124/510 (24.3%)	RR 0.75 (0.62–0.9)	61 fewer per 1000 (from 24 fewer to 92 fewer)	⊕⊕ΟΟ LOW	IMPORTANT
								20.2%		51 fewer per 1000 (from 20 fewer to 77 fewer)		

Quality assessment							No. of patients		Effect		Quality	Importance
No. of studies	Design	Risk of bias	Inconsistency	Indirectness	Imprecision	Other considerations	Awakening time (min)	Control	Relative (95% CI)	Absolute		
Awakening time												
5	Randomized trials	Serious*	Serious§	No serious indirectness	No serious imprecision	None	273	298	-	SMD 0.15 higher (0.02 lower to 0.31 higher)	⊕⊕ΟΟ LOW	IMPORTANT

* Lack of blinding in Hu C. 2021, Lan H. 2022, Gao, S. H. 2024; 3 study has a high risk of bias.

† Total number of events is <300.

‡ RR < 0.5.

§ I^2^ < 40%.

### 3.5. Results of meta-analysis

**Figure 3.**
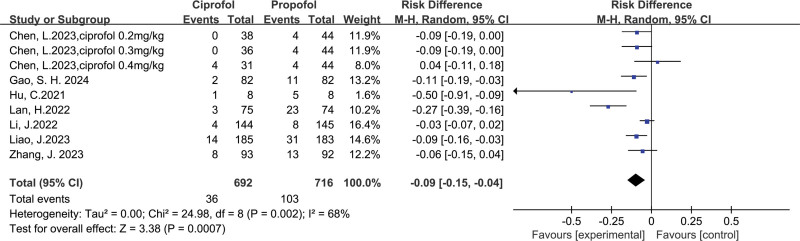
Results of the incidence of respiratory depression.

**Figure 4.**
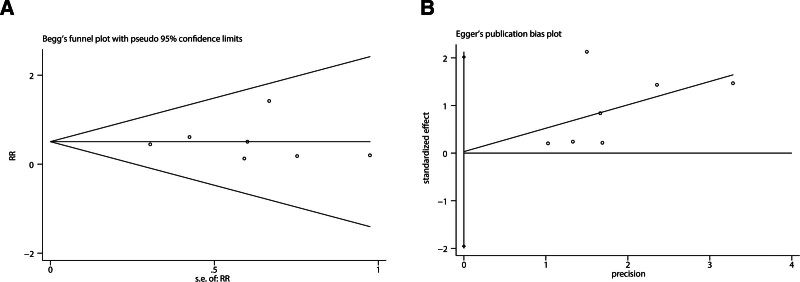
Results of the Begg test and Egger test.

Ciprofol versus propofol on respiratory depression: 7 trials,^[16–22]^ including 1408 patients, investigated the incidence of respiratory depression, by comparing ciprofol with propofol. The incidence of respiratory depression (pooled RD = −0.09, 95% CI: −0.15 to −0.04) in the ciprofol group was significantly lower than in the propofol group (Fig. 3). Begg test with *P* = .881 and Egger test with *P* = .969 suggested that no significant publication bias existed in the comparisons of respiratory depression between ciprofol with propofol (Fig. 4). Further, factors that affected respiratory depression were evaluated through subgroup analysis.

**Figure 5.**
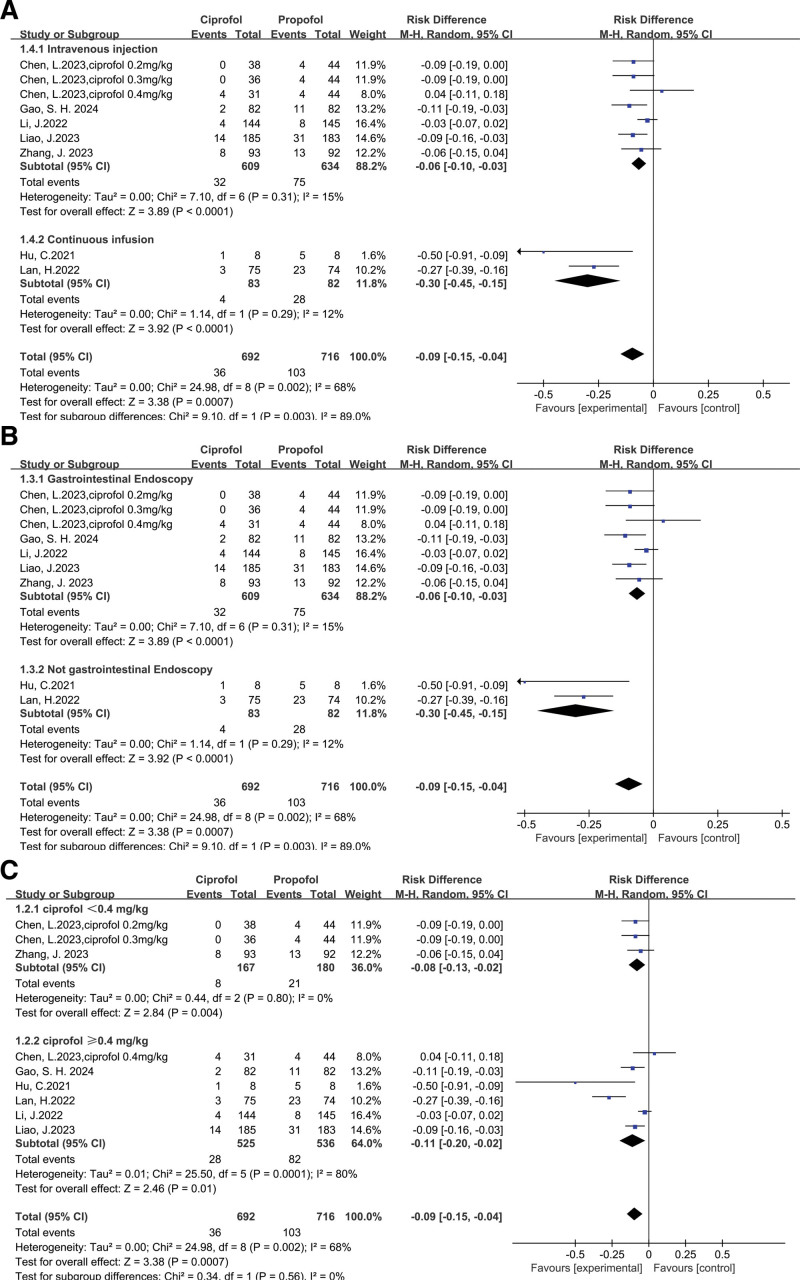
Results of subgroup analysis of the incidence of respiratory depression by method of administration (A), type of operation (B), and dosage of administration (C).

Method of administration: ciprofol significantly reduced the incidence of respiratory depression (pooled RD of 5 trials^[17,18,20–22]^: −0.06, 95% CI: −0.10 to −0.03) when the method of administration is intravenous injection, and also continuous infusion (pooled RD of 2 trials^[16,19]^: −0.30, 95% CI: −0.45 to −0.15) (Fig. 5A).

Type of operation: ciprofol significantly reduced the incidence of respiratory depression (pooled RR of 5 trials^[17,18,20–22]^: −0.06, 95% CI: −0.10 to −0.03) when the type of operation is gastrointestinal endoscopy, but also not gastrointestinal endoscopy (pooled RD of 2 trials^[16,19]^: −0.30, 95% CI: −0.45 to −0.15) (Fig. 5B).

Dosage of administration: Ciprofol significantly reduced the incidence of respiratory depression (pooled RD of 6 trials^[16–21]^: −0.11 95% CI: −0.20 to −0.02) when the dosage of ciprofol is more than or equal to 0.4 mg/kg, but also <0.4 mg/kg (pooled RD of 2 trials^[18,22]^: −0.08, 95% CI: −0.13 to −0.02) (Fig. 5C).

**Figure 6.**
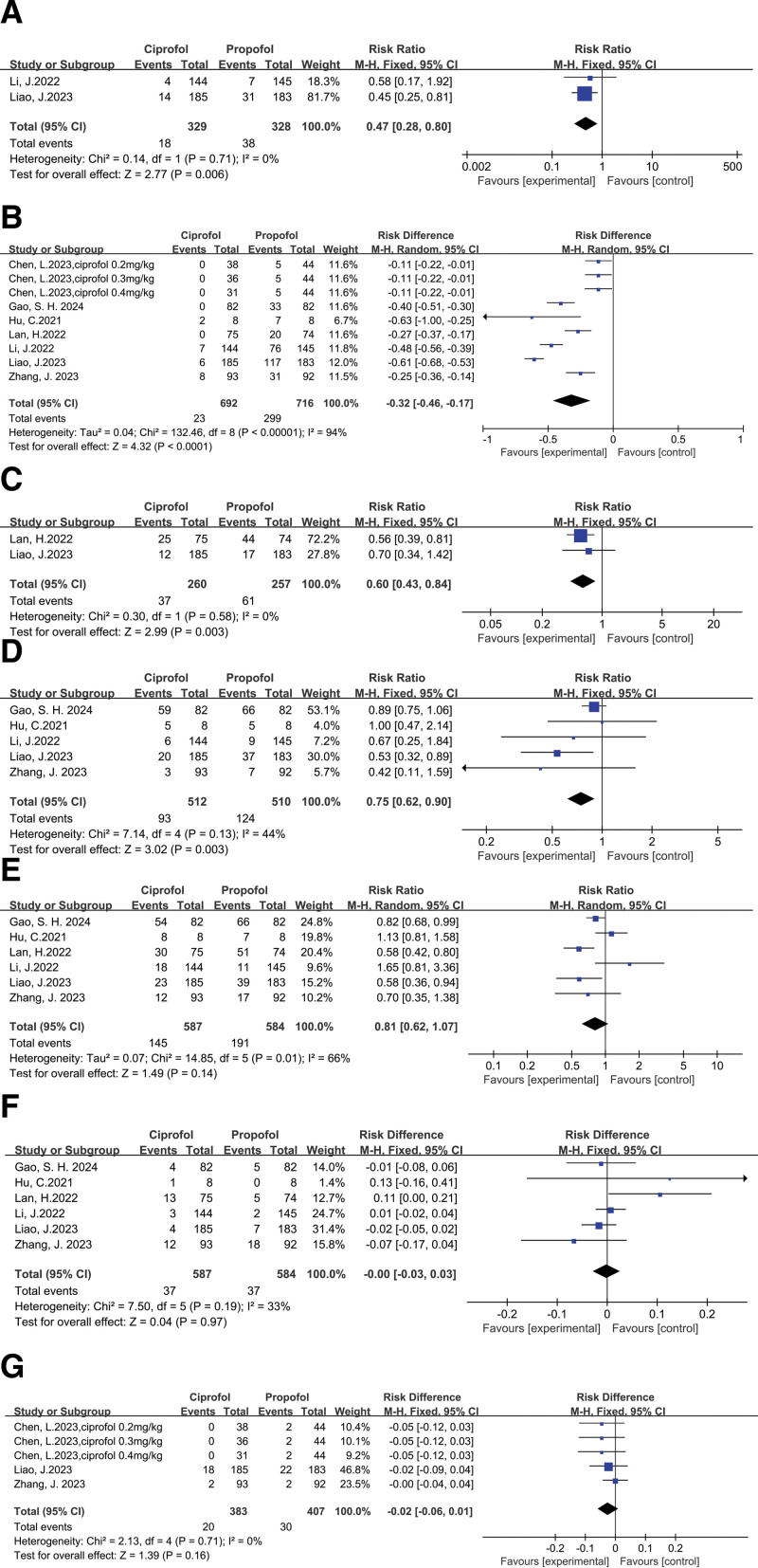
Results of the incidence of hypoxemia (A), injection pain (B), body movement (C), dizziness (D), hypotension (E), bradycardia (F), and PONV (G).

The incidence of hypoxemia, injection pain, body movement, dizziness, hypotension, bradycardia, PONV: ciprofol significantly reduced the incidence of hypoxemia (pooled RR of 2 trials^[17,20]^: 0.47, 95% CI: 0.28 to 0.80) (Fig. 6A), injection pain (pooled RD of 7 trials^[16–22]^: −0.32, 95% CI: −0.46 to −0.17) (Fig. 6B), body movement (pooled RR of 2 trials^[19,20]^: 0.60, 95% CI: 0.43 to 0.84) (Fig. 6C) and dizziness(pooled RR of 5 trials^[16,17,20–22]^: 0.75, 95% CI: 0.62 to 0.90) (Fig. 6D), meanwhile ciprofol almost reduced the incidence of hypotension (pooled RR of 6 trials^[16,17,19–22]^: 0.81, 95% CI: 0.62 to 1.07) (Fig. 6E) and PONV (pooled RD of 3 trials^[18,20,22]^: −0.02, 95% CI: −0.06 to 0.01) (Fig. 6G) without statistical significance compared with propofol, but ciprofol did not reduce the incidence of bradycardia (pooled RD of 6 trials^[16,17,19–22]^: −0.00, 95% CI: −0.03 to 0.03) (Fig. 6F).

**Figure 7.**
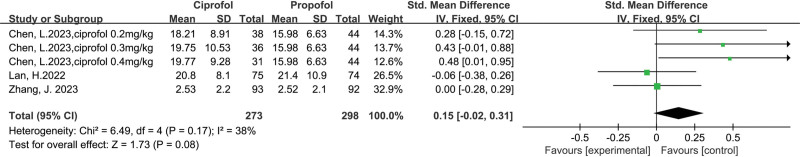
Results of awakening time.

Awakening time: ciprofol did not increase awakening time (pooled SMD of 3 trials^[18,19,22]^: 0.15, 95% CI: −0.02 to 0.31) (Fig. 7).

## 4. Discussion

Respiratory depression is a common problem after surgery. This meta-analysis aimed to assess the incidence of respiratory depression after anesthesia with ciprofol and propofol. The main results were as follows: (1) the incidence of respiratory depression in the ciprofol group was significantly lower than in the propofol group. (2) Ciprofol significantly reduced the incidence of respiratory depression when the method of administration is intravenous injection and continuous infusion. Meanwhile, ciprofol significantly reduced the incidence of respiratory depression when the dosage of ciprofol is more than or equal to 0.4 mg/kg and <0.4 mg/kg. Ciprofol significantly reduced the incidence of respiratory depression when the type of operation is gastrointestinal endoscopy, but also not gastrointestinal endoscopy. (3) Ciprofol significantly reduced the incidence of hypoxemia, injection pain, body movement, and dizziness, while ciprofol almost statistically insignificantly reduced the incidence of hypotension and PONV, but ciprofol almost statistically insignificantly increased the incidence of bradycardia. (4) Although ciprofol significantly increased the time to awakening, the time to awakening was <15 minutes.

Several studies have found that the decrease in oxygen saturation was less in the ciprofol group than in the propofol group, which indicates that ciprofol has less effect on respiratory depression.^[23]^ This meta-analysis also identified this consequence. Therefore, ciprofol is more suitable for patients than propofol. Compared with propofol, ciprofol has a lesser incidence of respiratory depression and might therefore be a potentially safer surgical option. If significant airway obstruction or respiratory arrest occurs during the procedure, the anesthesiologist will perform appropriate airway interventions, such as mask ventilation.^[20]^ We hypothesized that the reduction of respiratory depression by ciprofol may be related to central nervous system or airway collapse.^[16]^ However, this speculation requires further research shortly. Injection pain is one of the most common adverse effects of propofol administration, causing discomfort, increasing patient distress and anxiety, and leading to physical movement that can impede the successful completion of the procedure.^[24]^ The reported incidence of propofol injection pain varies widely, ranging from 30% to 70%. The incidence of injection pain of propofol has been reported to range from 30% to 70%. In the present meta-analysis, we also found that ciprofol significantly reduced the incidence of injection pain. 1% solution of ciprofol contained 5% soybean oil, 2.25% glycerol, and 1.2% purified egg phosphatidylinositol while 1% solution of propofol contained 10% soybean oil, 2.25% glycerol, and 1.2% purified egg phosphatidylinositol, and it is not difficult to find that the concentration of the drug in the aqueous phase of the emulsion is much lower in the ciprofol and therefore ciprofol caused less injection pain than propofol.^[25]^ Ciprofol has poor water compatibility and is therefore formulated as an oil-in-water emulsion. Compared to propofol, ciprofol is more hydrophobic and has a lower blood plasma concentration, which reduces injection pain. Compared to propofol, ciprofol significantly reduces the incidence of body movement, probably owing to the deeper level of sedation provided by ciprofol.^[19]^

In our meta, compared with the propofol group, the decrease of hypotension after anesthesia in the ciprofol group was smaller, suggesting that the inhibition of myocardial contractility and the expansion of peripheral blood vessels were less affected by ciprofol, which could provide a more stable hemodynamic environment for patients during the surgery.^[26]^ Ciprofol and propofol decreased HR, due to the inhibition of sympathetic nerve activity after anesthesia, resulting in the weakening of stress response. Ciprofol increased the incidence of bradycardia compared to propofol which may be caused by less injection pain and the incidence of cough.^[27]^

Considering all factors, ciprofol may be a better choice than propofol as an anesthetic, due to its minimal impact on blood pressure, with more stable hemodynamic changes during anesthesia.

Dizziness is a common adverse reaction. Its occurrence is related to gender, age, vestibular system, cardiovascular, and cerebrovascular diseases, and the use of narcotic drugs. Ciprofol reduced the incidence of dizziness which may be related to the smaller impact of ciprofol on hypotension and less residual accumulation, indicating that patients in the ciprofol group have better postoperative comfort.^[28]^

Research shows that propofol can prevent postoperative vomiting and nausea, and its mechanism may be related to its activation of GABAa receptors. Our meta shows that propofol and ciprofol all can reduce the incidence of PONV, the sedative mechanism of ciprofol may be also the activation of the GABA receptor. Although ciprofol did not significantly reduce the incidence of PONV, there was a trend which may be due to its affinity with the GABAa receptor being stronger.^[18]^

The terminal elimination half-life of ciprofol is similar to that of propofol, but the potency is approximately 5 times that of propofol, and the recovery time in patients with ciprofol is slightly longer. The reason for this may be the longer elimination half-life and lower clearance of ciprofol. However, although patients in the ciprofol group took longer to achieve full consciousness and to be discharged from the hospital, the lead time from the end of the procedure to discharge was <15 minutes, which is not clinically significant. However, further studies are needed to compare the pharmacologic properties of ciprofol and propofol.^[29]^

## 5. Limitations and suggestion for practice

Still, this meta-analysis has some limitations. First, heterogeneity and interference were caused by the fact that different surgeons used different sedation and anesthesia options for the post-sedation procedures. Second, heterogeneity due to age, especially between young and old people, had a significant impact. Third, some used analgesics and some did not, which also had an impact on the overall results. Fourth, most of the studies we included were from China, which may have led to poor extrapolation of findings and potentially large publication bias. Fifth, most of the trials were conducted in the American Society of Anesthesiologists (ASA) class I or II patients, but further studies are needed in elderly, frail, and critically ill patients. There is a lack of data on older adults with heart disease and respiratory disease. There is a lack of data on American Society of Anesthesiologists Class III.

## 6. Conclusion and recommendations

From this meta-analysis, it is demonstrated that ciprofol might reduce the incidence of respiratory depression and injection pain. These benefits are important in surgery to ensure safe and rapid postoperative recovery. So, ciprofol may be a safe and appropriate drug with fewer adverse effects used in clinical anesthesia.

## Author contributions

**Data curation:** Qian Cao, Jinjin Jian.

**Project administration:** Xiao Liang.

**Software:** Aonan Hong, Zhen Gu.

**Writing – original draft:** Jinfang Zeng.

**Writing – review & editing:** Aonan Hong.
